# Prescription of CRRT: a pathway to optimize therapy

**DOI:** 10.1186/s13613-020-0648-y

**Published:** 2020-03-06

**Authors:** Ayman Karkar, Claudio Ronco

**Affiliations:** 1 Medical Affairs-Renal Care, Scientific Office, Baxter A.G., Burj Al Salam, PO Box 64332, Dubai, United Arab Emirates; 2 Department of Nephrology Dialysis and Transplantation, International Renal Research Institute of Vicenza, San Bortolo Hospital, Vicenza, Italy

**Keywords:** Acute kidney injury, Sepsis, oXiris, Adsorption, Continuous renal replacement therapy, Anticoagulation, Vascular access

## Abstract

Severe acute kidney injury (AKI), especially when caused or accompanied by sepsis, is associated with prolonged hospitalization, progression to chronic kidney disease (CKD), financial burden, and high mortality rate. Continuous renal replacement therapy (CRRT) is a predominant form of renal replacement therapy (RRT) in the intensive care unit (ICU) due to its accurate volume control, steady acid–base and electrolyte correction, and achievement of hemodynamic stability. This manuscript reviews the different aspects of CRRT prescription in critically ill patients with severe AKI, sepsis, and multiorgan failure in ICU. These include the choice of CRRT versus Intermittent and extended hemodialysis (HD), life of the filter/dialyzer including assessment of filtration fraction, anticoagulation including regional citrate anticoagulation (RCA), prescribed versus delivered CRRT dose, vascular access management, timing of initiation and termination of CRRT, and prescription in AKI/sepsis including adsorptive methods of removing endotoxins and cytokines.

## Introduction

Continuous renal replacement therapy (CRRT) is a slow and smooth continuous extracorporeal blood purification, which is designed to replicate depurative function of the kidney [1, 2]. It is usually implemented over 24 h to several days with an aim of gentle correction of fluid overload and removal of excess uremic toxins. Untreated severe acute kidney injury (AKI) in critically ill patients is associated with high mortality rate. Renal replacement therapy (RRT) represents a better modality for the management of severe AKI. Though there is no clear evidence demonstrating the superiority of CRRT in survival rate to that of intermittent renal replacement therapy (IRRT), the CRRT superiority seems to find clear evidence when specific groups of patients are considered, and where a personalized treatment and modality is indicated. Furthermore, many observational studies considered CRRT as the predominant form of RRT in the intensive care unit (ICU) for critically ill patients with AKI and/or multiorgan failure (typically due to septic shock), along with acute brain injury or other causes of increased intracranial pressure or generalized brain edema. The effectiveness of CRRT is mainly due to its accurate volume control, steady acid–base and electrolyte correction, and achievement of hemodynamic stability in adults and pediatrics, where, in most cases, a clinical judgment has already been made that such patients cannot tolerate the relatively fast removal of fluids (and solutes) by conventional intermittent hemodialysis (HD) [3].

Continuous renal replacement therapy witnessed significant improvement since the technique was implemented by Peter Kramer of Göttingen (Germany) in 1977 [4]. The technique was established when Kramer was trying to introduce a catheter into the femoral vein for initiating HD. Although the catheter was inadvertently inserted into the femoral artery, Kramer realized that the patient’s mean arterial pressure could create a sufficient arteriovenous pressure difference in the extracorporeal circuit to drive blood flow. This fortuitous occurrence was the genesis of continuous arteriovenous hemofiltration (CAVH), which provided net convective solute removal (depuration) through the combination of ultrafiltration and plasma volume restitution with replacement solution. Later, in 1987, Peter Robert Uldall (Toronto, Canada) [5] introduced “continuous veno-venous hemofiltration (CVVH)” by incorporating a pump and replacing the dependence of blood flow rate on the patient’s mean arterial pressure. This technique avoided not only the potential risks and complications of puncturing a major artery (e.g., infection, distal thrombosis, and disconnection/bleeding), but also the unpredictability of extracorporeal blood flow rates due to changes in a patient’s hemodynamic status.

## Prescription

The success of CRRT depends on the prescribed and achieved doses, which are a function of fluid replacement and/or dialysate administration rates, treatment duration, type of dialyzer and method, and dose of anticoagulation. Moreover, delivery and performance of CRRT require a well-established protocol (e.g., indications for initiation/cessation, catheter management) and well-trained medical and nursing staff [6].

### CRRT versus intermittent and extended HD

Patients with severe AKI in the ICU usually require RRT in the form of intermittent HD (IHD), peritoneal dialysis (PD), extended HD (slow low-efficiency dialysis: SLED), or CRRT. The current evidence and KDIGO guideline support the implementation of CRRT in hemodynamically unstable patients and those with increased intracranial pressure [7]. Moreover, there is increasing evidence that CRRT is associated with a trend of short- and long-term dialysis independence [8–11]. However, there is no evidence demonstrating a mortality difference between all these modalities of RRT [8, 10]. The KDIGO guideline recommends the use of CRRT and IHD as complementary therapies in AKI patients [7].

### Life of the filter/dialyzer

Premature (non-elective) termination of CRRT is usually due to clotting in the extracorporeal circuit, most commonly the filter. Filter clotting is associated with blood loss, inadequate dialysis due to treatment interruption, and increased costs related to set utilization. The major causes of short-lived circuits are inadequate anticoagulation, high filtration fraction (FF), and a compromised vascular access, which may result in stagnation of blood in the extracorporeal circuit while frequent machine alarms are managed.

Filtration fraction (FF) is the ratio of net plasma water removal rate to the plasma flow rate delivered to the filter. The official definition of FF is *“the percentage ratio of ultrafiltration rate to plasma flow rate, where plasma flow rate equals blood flow rate X (1*-*hematocrit)”.* Practically, FF should not exceed 20–25%—higher FFs correspond to higher post-filter hematocrit, which promotes clot formation and degradation of filter performance [12].

### Anticoagulation

The aim of anticoagulation is to maintain patency of the extracorporeal circuit while minimizing patient complications. Appropriate anticoagulation is a subtle balance between clotting and bleeding. Strategies to prevent clotting include general measures, such as saline flushes and online pre-dilution, and different anticoagulants, such as unfractionated and low molecular weight heparin, heparin coated membranes (e.g., oXiris), and regional citrate anticoagulation (RCA).

Heparins are the most widely used anticoagulants in continuous renal replacement procedures. They are widely available and can be easily monitored but have some disadvantages. These include risks of hemorrhage, heparin resistance, and heparin-induced thrombocytopenia (HIT). The safety and efficacy of heparin therapy are based on monitoring the activated partial thromboplastin time (APTT), which is a good predictor of the risk for filter coagulation and patient hemorrhage. It is recommended to adjust the systemic APTT between 35 and 45 s [13].

Regional citrate anticoagulation has been shown to be safe and effective for anticoagulation of CRRT in most ICU patients [14]. It is based on the ability of citrate to prevent coagulation in the extracorporeal circuit by binding and chelating free ionized calcium, which is a critical co-factor in both the intrinsic and extrinsic coagulation cascades. One molecule of citrate binds two calcium anions forming citrate–calcium complex. Based on the specific CRRT modality and other factors, including flow rates and membrane surface area, approximately 60% of this complex is lost in the CRRT effluent, due to its low molecular weight (298 D). Some complexes, however, are delivered by the venous blood line to the systemic circulation and are metabolized in the liver, where one citrate molecule is transformed into three bicarbonate molecules, and calcium is released into the circulation. However, because this amount of released calcium does not completely replace the calcium lost in the effluent, calcium is infused through a separate central blood line to maintain systemic (blood) ionized calcium in the normal range (1.1–1.3 mmol/L). Initially, circuit and patient ionized calcium levels are measured frequently, but once stable doses are documented they are measured every 6–8 h [15]. In the presence of severe liver failure, citrate accumulation may occur and is best detected by the total Ca^++^/iCa^++^ ratio. A ratio of > 2.5 indicates citrate accumulation syndrome and treatment must be stopped [16]. The dialysate and replacement solutions should be calcium-free to avoid interaction and reduction of the anticoagulation effect. RCA requires close monitoring, especially upon initiation of therapy, and management of RCA can prevent some possible side effects, such as metabolic alkalosis (1 mmol of citrate converted to 3 mmol of HCO_3_ in the liver), metabolic acidosis (citrate can accumulate if there is liver or skeletal muscle dysfunction), hypocalcemia and hypercalcemia (inadequate control of chelating calcium by citrate or excess infusion of calcium), hypernatremia (when hypertonic trisodium citrate is used), and hypomagnesaemia (from binding to citrate–Ca^2+^ complex). However, RCA has been associated with significantly less bleeding [17], less blood transfusion [18] and extended life of the extracorporeal circuit [19]. Generally, anticoagulation for CRRT should be adapted to the patient’s characteristics and institution’s experience. The KDIGO guidelines suggested using RCA rather than heparin in patients who do not have contraindications for citrate [7].

### CRRT dose

CRRT dose may be represented by the volume of blood purified per unit of time and is quantified by effluent rate normalized to body weight (unit: mL/kg/h). In clinical practice, effluent comprises net ultrafiltrate (according to net fluid removal requirements) along with replacement fluid and/or dialysate, depending on the specific CRRT modality.

In a seminal 2000 publication, Ronco et al. [20] demonstrated that prescribed doses of 35 and 45 ml/kg/h in post-dilution hemofiltration were superior to 25 ml/kg/h with respect to survival, resulting in a 15–20% absolute increase and an approximately 40% relative increase among critically ill AKI patients. However, later studies of higher prescribed doses of 48 versus 20 ml/kg/h in post-dilution CVVH [21] and 35 versus 20 ml/kg/h in pre-dilution CVVHDF [22] showed no difference in survival rates. These studies were followed by three major multicenter randomized controlled trials: ATN-CVVHDF, 20 versus 35 ml/kg/h pre-dilution CVVHDF in USA [23]; RENAL-CVVHDF, 25 versus 40 ml/kg/h post-dilution CVVHDF in Australia and New Zealand [24]; and IVOIRE-CVVHF in France, Belgium and Netherlands, 35 versus 70 ml/kg/h combined pre/post-dilution hemofiltration [25], which confirmed that increasing dose intensity above 20–25 ml/kg/h does not improve survival in critically ill patients with severe AKI. Furthermore, two meta-analyses have evaluated CRRT dose effects in AKI. Van Wert et al. [26] assessed 12 studies with 3999 patients and showed no benefit of more intensive RRT with regard to survival or dialysis dependence among survivors. Clark et al. [24] evaluated high-volume hemofiltration (> 50 ml/kg/h) for septic AKI patients, and found no difference in mortality between high-dose and standard-volume hemofiltration, but significantly higher rates of hypophosphatemia and hypokalemia in high-volume hemofiltration-treated patients. Other studies have confirmed the higher rates of hypophosphatemia, hypokalemia, loss of amino acid or protein, vitamins, selenium, and folic acid—concern has been raised especially about inappropriately low concentrations of water-soluble antibiotics, especially in septic patients [27, 28]. In addition, the RENAL study found that higher dose post-dilution CVVHDF resulted in greater filters’ consumption, indicating more clotting events and frequent interruption occurred during therapy [25].

A common oversight in clinical practice is failure to differentiate between prescribed and delivered dose. Interruption of CRRT treatment, due to circuit clotting, machine alarms, change of replacement solutions, radiological investigations and/or surgical procedures, can have substantial impact on the actual delivered dose. The KDIGO clinical practice guideline recommends the following [7]:

- “*In clinical practice, in order to achieve a delivered dose of 20*–*25* *ml/kg/h, it is generally necessary to prescribe in the range of 25*–*30* *ml/kg/h, and to minimize interruptions in CRRT*”.
- “*The dose should be frequently assessed, and prescription should be adjusted accordingly*”

### Vascular access

A properly functioning vascular access is critical in delivering the prescribed dose of CRRT. An inadequate vascular access may not be able to provide sufficient blood flow to achieve treatment goals, especially in convective therapies. A very common occurrence is the development of high (negative) arterial and/or venous pressures when the blood pump attempts to deliver the prescribed blood flow rate to the circuit. If relatively simple interventions (i.e., repositioning the catheter or the patient) do not correct this problem fairly promptly, a prolonged period of blood stagnation in the circuit occurs, potentially leading to clotting, treatment interruption, and circuit loss. Proper nursing management of catheters is crucial, not only in avoiding these complications but also in ensuring that appropriate hygienic measures are taken to minimize infection risk.

The location of the vascular access should be dictated by blood flow rate requirements, the anticipated duration of use, and ease of placement according to patient-related attributes (e.g., obesity). As suggested previously, convective modalities (especially CVVH) require higher blood flow rates than CVVHD due to filtration fraction constraints in post-dilution and dilution-related efficiency impairment in pre-dilution. For doses currently recommended in clinical practice, blood flow rates of less than 200 mL/min are generally required in CVVH (irrespective of dilution mode), especially in larger patients. Parienti et al. [29] showed that, in a randomized controlled trial evaluating catheter dysfunction and dialysis performance according to vascular access among 736 critically ill adults, catheter survival is best with a right internal jugular vein location, followed by femoral vein and left internal jugular vein. The KDIGO AKI guideline [7] recommended the sites of catheter placement by order of preference: right internal jugular vein > > femoral vein > left internal jugular vein > subclavian vein (dominant side) > subclavian vein (non-dominant side).

Another consideration is catheter diameter, which is dependent on RRT modality. For example, in CVVHD modality, where blood flow rate ranges from 100 to 150 ml/min (though it is highly dependent on the rate of dialysate and the selected membrane), 11–12 French size is recommended. On the other hand, for CRRT performed in conjunction with extracorporeal CO_2_ removal (requiring a high blood flow rate of 400–500 ml/min), 14–16 French size is required [12].

Vascular access can be a major challenge in neonates and infants. Large-diameter catheter has been recommended in pediatric CRRT, as it has been associated with better functional survival of the CRRT circuit especially when the catheter is placed in the internal jugular vein versus the subclavian or femoral vein. Selection of catheter size (double- or triple-lumen) depends on patient’s body size, which varies from 7 to 12 French size for neonates of 3–6 kg to children of more than 30 kg body weight [30]. Unlike vascular access placement for long-term hemodialysis, the vascular access for pediatric CRRT is often placed percutaneously, without a cuff, and at the bedside [31]. When catheters are not in use, they are routinely locked with high-concentration heparin solutions, but not in small infants.

### CRRT initiation and termination

The optimal time of initiation and termination of CRRT remains controversial. In this regard, a major problem is the lack of a definition of timing and what constitutes “early” versus “late” initiation. For example, should initiation be based on onset of symptoms, biomarkers thresholds, time measured relative to the onset of AKI or ICU admission, or AKI classification criteria? There are numerous perspectives but no broad consensus to guide clinicians. The KDIGO AKI guideline [7] recommends the following:

- “*Initiate RRT emergently when life*-*threatening changes in fluid, electrolyte, and acid*–*base balance exist*”
- “*Consider the broader clinical context, the presence of conditions that can be modified with RRT, and trends of laboratory tests, rather than single BUN and creatinine thresholds alone, when making the decision to start RRT*”.

In 2016, two large prospective randomized controlled trials (RCTs) assessed the impact of different renal replacement therapy timing in severely ill ICU patients with AKI without potentially life-threatening complications. These are the Artificial Kidney Initiation in Kidney Injury (AKIKI) study, a multi-center trial performed in France, and the Early versus Late Initiation of Renal Replacement Therapy in Critically Ill Patients with AKI (ELAIN) trial, a single-center German study. The AKIKI trial, conducted in 620 ICU patients on mechanical ventilation and/or catecholamine infusion with KDIGO stage 3 AKI, showed no significant difference in 60-day mortality was found between early and delayed RRT [32]. However, a post-hoc analysis, in which patients who did not ultimately receive RRT were treated as a separate group, showed late start was associated with significantly higher mortality in comparison with the early group.

The ELAIN trial, conducted over a similar time period, screened 604 almost exclusively postsurgical and trauma patients to include 231 ICU patients with KDIGO stage 2 AKI with a plasma NGAL level > 150 ng/ml. An early initiation strategy resulted in lower 90-day mortality, more rapid recovery of renal function, and a significantly shorter duration of hospital stay [33]. Longer term (12 months) follow-up showed a persistent beneficial effect on survival rate in the group who received early versus late start CRRT. These findings require confirmation in larger, multicenter, RCTs involving different patient groups requiring RRT. Following these two studies, several meta-analysis studies comparing early and late CRRT initiation were published with conflicting results [34–37].

The most recent timing RCT involved either an early group who received RRT within 12 h after documentation of failure-stage AKI or a late group who received RRT after a delay of 48 h if renal recovery had not occurred [38]. This French study, which assessed the primary outcome as death at 90 days, showed no difference between early versus late start of CRRT. However, one major limitation of this trial was the reliance for timing decisions on the RIFLE classification system, which has been shown to be relatively insensitive for the purpose in comparison to other approaches. The second limitation is the choice of a delay period of only 48 h, which may not be sufficiently long to allow recovery of renal function or detect a difference between early and delayed RRT [38].

More recently, the Cochrane Database of Systematic Reviews studied the timing effect of RRT initiation for AKI on death (day 30 or after 30 days) and recovery of renal function according to modality of RRT (continuous RRT vs continuous and intermittent RRT), etiology of AKI (surgical vs non-surgical), clinical–biochemical criteria, length of stay in ICU, and adverse effects. This review, which included five randomized studies enrolling 1084 participants, concluded that early RRT may reduce the risk of death and may improve the recovery of kidney function in critically patients with AKI. However, there was an increased risk of adverse events with early RRT [11].

Finally, the acute disease quality initiative (ADQI) workgroup suggested that a more personalized approach can be developed based on the dynamic assessments of different clinical parameters that reflect the mismatch of demand and capacity [39]. While specific parameters quantifying demand and capacity have not been defined, this “precision” approach merits further assessment.

Discontinuation of CRRT, as defined by the KDIGO AKI guideline, is “*when RRT is no longer required either because intrinsic kidney function has recovered to the point that it is adequate to meet patient needs, or because RRT is no longer consistent with the goals of care*”. These guidelines also state that “*using diuretics is not recommended to enhance kidney function recovery*, *or to reduce the duration or frequency of RRT*” [7]. Recent studies showed that improvement in urine output and daily urinary creatinine evaluation remain reliable markers of renal recovery [40]. Clinical indicators, more specific to discontinuation of CRRT, include vasopressor cessation, increased urinary output ≥ 500 ml/24 h (without diuretics), hemodynamic stability, correction of fluid overload, and the possible need to shift to IHD due to imminent discharge from the ICU.

### Prescription in sepsis

In 2016, The Sepsis Definitions Task Force of the Society of Critical Care Medicine/European Society of Intensive Care Medicine published a revised definition of sepsis in which sepsis was defined as life-threatening organ dysfunction caused by a dysregulated host response to infection [41]. Septic shock was defined as a subset of sepsis in which underlying circulatory and cellular/metabolic abnormalities are profound enough to substantially increase mortality.

Studies have reported that approximately 10% to 40% of ICU patients have sepsis [42–44], and approximately 25% to 30% of patients develop sepsis within 24 h of ICU admission [45–47]. The mortality of patients with sepsis in the ICU varies from 15 to 35% [48]. Furthermore, sepsis is the primary cause of AKI in the ICU and is associated with short- and long-term adverse outcomes along with increased risk of death. Renal dysfunction/failure has been reported to occur in 22% to 69% of patients with sepsis [49, 50]. In sepsis-associated AKI, 15–20% of patients develop severe stages of renal insufficiency and need RRT [51–54].

Untreated severe AKI in critically ill patients is associated with high mortality. RRT represents the cornerstone of the management of severe AKI, and CRRT has been considered as the predominant form of RRT in the ICU, especially in AKI with sepsis [55]. This is due to its ability to provide accurate volume control, slow correction of metabolic abnormalities, and hemodynamic stability [7, 56, 57]. In addition, CRRT has been shown to be associated with lower rate of dialysis dependence than intermittent hemodialysis [8–11].

Patients with sepsis typically have elevated blood levels of endotoxins [58] and cytokines [e.g., interleukin 6 (IL-6), IL-8, IL-10, and tumor necrosis factor alpha(TNF-α)] [59]. Increased plasma levels of endotoxin can be the result of either infection due to Gram-negative bacteria or the translocation of endotoxin from Gram-negative bacteria across the wall of the abnormally permeable gut [60, 61]. Levels of cytokines vary considerably by patient and cytokine, and by factors such as sepsis severity and time since diagnosis or ICU admission [62, 63]. A retrospective cohort study of the cytokine response to pneumonia found that levels of IL-6, IL-10, and TNF-α were significantly higher among those who developed severe sepsis (survivors and non-survivors) compared with those who did not [64]. Elevated levels of IL-6 are usually associated with an increased risk of mortality [65, 66] or post-discharge mortality [67, 68].

Removal of endotoxins and plasma cytokines by extracorporeal blood purification devices may control the associated dysregulation of the immune system (which is known to induce organ dysfunction) and is potentially associated with improved outcomes in patients with sepsis [69]. A meta-analysis of randomized controlled trials of patients with sepsis or septic shock found that when all types of extracorporeal blood purification were combined, the risk of hospital, 28-day, and overall mortality was reduced compared with no blood purification [70]. However, as mentioned previously, high-volume CVVH (IVOIRE, 70 ml/kg/h) did not improve clinical outcomes in septic AKI patients (vs a dose intensity of 35 ml/kg/h). Likewise, a systematic review/meta-analysis performed by Clark et al. [71] failed to find a clinical benefit for high-volume hemofiltration (> 50 ml/kg/h) in septic AKI patients. Removal of endotoxins and plasma cytokines by plasma exchange showed conflicting results in the reduction of mortality in patients with sepsis or septic shock [72, 73].

Recent studies using different types of membrane adsorbers of endotoxin and cytokines have shown some promising results (Table 1). Some clinical improvement was reported in selected studies involving septic patients treated with hemoperfusion devices comprised polymyxin B-bound fibers designed to adsorb endotoxin [74–79]. However, other studies showed no difference in clinical endpoints between polymyxin B plus conventional therapy and conventional therapy alone [80–83]. Endotoxin removal by polymyxin B is thought to occur by a combination of ionic and hydrophobic interactions.

**Table 1:** Comparison between three different types of filters used in AKI/CRRT with sepsis

Specifications	Toraymyxin (Toray industries)	CytoSorb (Cytosorbents)	oXiris (Baxter)
Membrane material	PS-based composite woven fiber with immobilized polymyxin B	PSDVB copolymer beads, which are highly porous and covered with PVP	Copolymer of AN69-coated with PEI and unfractionated heparin
Structure	Woven fibers	Microporous beads	Hollow fiber, symmetric
Sterilization mode	Steam	Gamma irradiation	EtO
Adsorptive capacity	Endotoxins	Cytokines	Endotoxin and cytokines
Performance of CRRT	No	No	Yes
Heparin-grafted inner surface	No	No	Yes Grafted (immobilized) heparin, which imparts an anti-thrombogenic characteristic
Mode of adsorption	Ionic and hydrophobic interactions	Hydrophobic interactions	• Adsorbing cytokines through ionic interactions due to its content of sulfonate groups • Adsorbing endotoxins through ionic interactions due to its high concentration of PEI on the inner aspect of the membrane

*AN69* acrylonitrile and methalylsulfonate, *EtO* ethylene oxide, *PEI* polyethylenimine, *PSDVB* polystyrene divinylbenzene, *PS* polystyrene, *PVP* polyvinylpyrrolidone

CytoSorb, a hemoperfusion device intended to remove cytokines by hydrophobic interactions, was employed recently in a small pilot study of patients with refractory septic shock. In this trial, a reduction in vasopressor dose compared to baseline in patients treated with the device was reported [84], although the results were difficult to interpret due to the lack of a control group. Moreover, an RCT [85] and a systematic review [86] found insufficient evidence to support a beneficial effect of CytoSorb on patient outcomes. Further, Schadler et al. did not find that CytoSorb achieved a significant reduction in plasma levels of IL-6 (vs conventional management), which may be necessary for improving patient outcomes. More recently, CytoSorb treatment in septic patients undergoing RRT showed a reduction in plasma levels of IL-8 but not of other cytokines. This study (of 24 h duration) showed an improvement of microcirculation despite no significant variation in macro-hemodynamics [87].

Clinical studies in patients with sepsis or septic shock using filters composed of the AN69 membrane, which is capable of adsorbing cytokines through ionic interactions due to its content of sulfonate groups, showed a reduction in vasopressor requirements [88], a decrease in lactate level after 3 h, and an increase in mean arterial pressure after 24 h of therapy [89]. These changes were accompanied by decreases in blood levels of TNF-α, IL-1β, IL-6, IL-8, IL-10, and HMGB1 after 72 h [89]. A registry study of CRRT in ICU patients (48% with sepsis) found that use of AN69 was associated with a reduction in mortality and ICU length of stay [90]. In later years, AN69 membrane was further developed and its surface was treated with polyethyleneimine (PEI) to produce AN69ST, which not only improved its biocompatibility but also offered the possibility to adsorb endotoxins through ionic interactions. The latest AN69-based generation of sets is oXiris, which has two unique features. One, enabled by the high concentration of PEI in the inner aspect of the membrane, is the increased potential to adsorb endotoxins from the blood. The other unique feature is grafted (immobilized) heparin, which imparts an anti-thrombogenic characteristic. Thus, in addition to its ability to perform CRRT treatment with an anti-thrombogenic membrane, oXiris has been shown to adsorb endotoxins and cytokines [55].

A retrospective case series of patients with septic AKI found that use of the oXiris set for CVVH was associated with a greater reduction in SOFA score after 48 h compared to historical controls. However, there was no difference between groups in vasopressor dose, ICU or hospital length of stay, or ICU or hospital mortality [91]. A recent Swedish clinical case report, in two patients with severe Gram-negative septic shock (KDIGO class 3 AKI) and verified endotoxemia, confirmed the ability of oXiris membrane to effectively adsorb endotoxins [92], thus corroborating earlier in vitro studies [93]. In another recent case report, a critically ill patient with severe sepsis secondary to a Gram-negative bacterial infection and sepsis-associated AKI was treated with CVVHDF using the oXiris set electively changed every 12 h for 3 consecutive days. The patient, who required high-dose vasopressor support and mechanical ventilation, showed reduction in the need for vasopressor support—by day 4, vasopressor requirements ceased and extubation occurred [94]. Similar findings were documented in a French study involving 31 septic patients receiving oXiris-based CRRT between 2014 and 2019. In this study, a lower hospital mortality than predicted (based on severity score) for the most critically ill patients was reported, although no improvement in the SOFA score over 48 h was observed. Nevertheless, there was an 88% relative decrease in norepinephrine infusion (with stabilization of hemodynamic status) and a significant improvement in lactatemia and pH over time. These improvements were most evident in patients with intra-abdominal sepsis and those with Gram-negative bacilli infection [95].

In a larger recent study, the medical records of 60 septic patients treated with CRRT using the oXiris set from April 2011 to December 2018 were reviewed—85% of patients had AKI. The results of this study, in which the use of oXiris was reported to be safe without adverse effects, showed improvement in cardio-renal and respiratory parameters, a decrease of the noradrenaline dosage, and a reduction in endotoxin activity assay, cytokines and procalcitonin [96].

Similar findings were recently documented in a prospective, randomized crossover, double-blind study of 16 patients, conducted between February 2016 and February 2018. These patients required CRRT for documented or suspected Gram-negative septic shock (blood endotoxin levels > 0.03 EU/mL) and KDIGO stage 3 AKI [97]. The patients received a total of 48 h of pre-dilution CVVHDF, 24 h each with the oXiris filter, and an AN69ST filter in random order. Endotoxin and cytokine levels were measured at baseline and at 1, 3, 8, 16, and 24 h after the start of each treatment period. Mean arterial pressure, norepinephrine infusion rate, and lactate levels were also recorded. Endotoxin level decreased more in the oXiris group than in the AN69ST group at 3, 8, and 16 h, which was accompanied with more substantial reduction in the cytokines TNF-α, IL-6, IL-8, and IFN-γ than in the standard filter group. oXiris treatment was associated with a favorable hemodynamic effect, as suggested by a more rapid decrease in blood lactate levels and lower doses of norepinephrine needed to maintain the mean arterial pressure.

Finally, a recent report included four sepsis-induced AKI patients who received CRRT with oXiris in ICUs of major Chinese hospitals. The aim of this study was to gain insights into the use of oXiris membrane for the stabilization of critically ill patients, which included start and end times, mode, dose, and additional anticoagulant. The authors concluded recommendations that oXiris should be considered for early treatment of septic AKI patients as an adjunctive therapy [98].

## Conclusions

Severe AKI, especially when caused by or associated with sepsis, carries increased risk of progression to chronic kidney disease and end-stage renal failure. In addition, it is associated with prolonged hospitalization and increased mortality rate, along with being a significant public health burden from financial perspective. Critically ill patients with AKI and/or multiorgan failure in ICU require special modalities of therapies in an attempt to achieve hemodynamic stability, euvolemic status, and acid–base and electrolytes balance with an aim of speeding up renal recovery and avoiding deleterious consequences. The superiority of CRRT seems to find clear evidence when specific groups of patients are considered. This is exactly the reason why physicians should be aware of the possibility to prescribe a personalized treatment and modality. In comparison to conventional intermittent HD, CRRT provides slow and relatively gentle treatment, and is indicated in hemodynamically unstable and brain edema patients. The prescribed dose should be 20–25 ml/kg/h, but to deliver this dose, higher doses are required. To avoid degradation of filter performance due to hemoconcentration, filtration fraction should not exceed 20–25%. The recommended method of anticoagulation is RCA. The preferred location for catheters placement is the right internal jugular vein, followed by the femoral vein and left internal jugular vein. CRRT management also includes consideration of proper timing of initiation and termination—prospective trials have provided conflicting results. While early initiation may have better survival and renal recovery rates, it may be associated with more complications. Finally, although clinical data are relatively scant and also conflicting, removal of endotoxins and cytokines in different settings of sepsis (with or without AKI) may have a positive impact on clinical outcomes. The oXiris hemofilter has been shown to be effective in adsorbing endotoxins and cytokines in laboratory and clinical studies, in addition to its capability of performing CRRT with a membrane having anti-thrombogenic properties.

## Data Availability

Not applicable.
